# CLINICIANS’ PERSPECTIVES ON THE EVOLUTION OF PALLIATIVE CARE DELIVERY DURING THE COVID-19 PANDEMIC

**DOI:** 10.1093/geroni/igac059.2990

**Published:** 2022-12-20

**Authors:** Laura Quintero Silva, Andiara Schwingel, Julie Bobitt, Minakshi Raj

**Affiliations:** University of Illinois Urbana-Champaign, Champaign, Illinois, United States; University of Illinois, Urbana Champaign, Urbana Champaign, Illinois, United States; University of Illinois at Chicago, Chicago, Illinois, United States; University of Illinois Urbana Champaign, Champaign, Illinois, United States

## Abstract

Palliative care has been primarily delivered to patients in person since its inception. During the Covid-19 pandemic, providing palliative care was especially challenging for clinicians due to public health measures to contain the virus that required them to interact virtually with patients and families. While the rapid implementation of telehealth has been examined in other clinical contexts, limited research has studied the impact of the pandemic on palliative care delivery. This study examined the experiences clinicians faced when providing palliative care to older adult patients during the pandemic. Between April 2021 and March 2022, we interviewed 29 geriatricians and palliative care specialists from 11 institutions across the US. We asked clinicians about their experiences with palliative care during the pandemic, including challenges and opportunities related to the changing nature of palliative care delivery. We analyzed interviews using reflexive thematic analysis. The following three themes emerged from the analysis: (1) Clinicians’ challenges adjusting to virtual care; (2) System-level barriers, restrictions, and uncertainties about Covid-19; and (3) Older adult patients’ context and vulnerability (i.e., loss of social engagement, isolation, loneliness, delayed access to care) that increased the complexity of their health conditions. In conclusion, clinicians’ experiences during the pandemic shed light on the evolution of palliative care delivery and the importance of preparing them for new care models that account for virtual delivery and that address the diverse needs of older adults that emerge during public health crises.

